# Social support and management of hypertension in south-west Nigeria

**DOI:** 10.5830/CVJA-2014-066

**Published:** 2015

**Authors:** Pauline E Osamor

**Affiliations:** Institute of Child Health, College of Medicine, University of Ibadan, Ibadan, Nigeria

**Keywords:** hypertension, social support, chronic disease, compliance, Nigeria, Africa

## Abstract

**Introduction:**

Social support can facilitate compliance or adherence to recommended treatment regimens, especially for chronic disease management. There is little data from Africa on the role of social support in the management of chronic disease.

**Objective:**

The current study investigated the relationship between social support for treatment compliance among hypertensive subjects in a poor urban community in southwest Nigeria. A second objective was identifying the correlates of social support in the study sample.

**Methods:**

The study was a community-based, cross-sectional and descriptive study of 440 community residents (mean age 60 years, 65.2% women) from Idikan community, Ibadan, Nigeria who had hypertension.

**Results:**

Most subjects (~ 93%) reported receiving some social support from family members and approximately 55% reported receiving social support from friends. Social support from friends (*p* < 0.0001) but not from family (*p* = 0.162) was significantly associated with good compliance with treatment for hypertension. Factors associated with receiving significant support from both family and friends included marital status and religion, while age and educational level were associated with receiving significant support from family members only. Gender was not significantly associated with receiving social support.

**Conclusion:**

We concluded that social support is strongly associated with hypertension treatment compliance in this community in south-west Nigeria. These findings suggest a need for exploring the promotion of social support as a useful tool in chronic disease treatment programmes.

## Abstract

The relationship between social support and health has been of great scientific interest for many years. Several epidemiological studies have pointed out the importance of social support for morbidity and mortality.^1^ For mortality, there are consistent indications of a lower risk of death among people with a large social network.^2^,^3^ This beneficial effect is confirmed for several morbidities, including cancer, coronary heart disease and other cardiovascular diseases (CVDs).^4^,^5^

Over the past quarter of a century, much research has convincingly documented the relationships between social networks and social support on morbidity, mortality, and a variety of positive chronic illness outcomes.^6^,^7^ A number of behaviours or mechanisms may modulate the relationship between social support and self-management. For example, it is reasonable to assume that family members and friends may facilitate the self-management process in a variety of ways, providing, for example, occasional advice, emotional support, tangible support that indirectly facilitates self-management (e.g. shopping for heart-healthy food), and more direct assistance with illness-management activities.

There is some evidence that illness-specific support is more predictive of health outcomes than general support.^8^ Therefore one might hypothesise that in the case of chronic illness selfmanagement, illness-specific or regimen-specific support may have a stronger influence on self-management behaviour than more global types of support.

Rozanski, Blumenthal and Kaplan^9^ reviewed 15 studies and found that people who reported low levels of social support were at greater risk of developing CVD. Blazer^10^ published similar findings, indicating that low levels of perceived social support were found to be risk factors for developing cardiac events. Other research has suggested that adherence to drug therapy was strongly associated with family support provided to patients with hypertension.^11^

Hypertension is a major public health problem and a major risk factor for stroke, cardiac failure and chronic renal disease in developing countries. Currently, one-quarter of the world’s adult population has hypertension, and modelled projections indicate an increase to 1.15 billion hypertensive patients by 2025 in developing countries.^12^

Several studies have examined the factors influencing compliance behaviours with hypertensive treatment. Among these studies, Marin-Reyes and Rodriguez-Moran^11^ found that compliance with hypertensive treatment was directly linked to the support of family members.

It has been well documented that patients from disrupted or isolated social circumstances are less likely to be good compliers than those with stable families and/or helpful friends. However, only recently have there been systematic studies of attempts to engender or direct social support in order to improve compliance with antihypertensive therapy. These studies have not shown an independent effect on compliance of attempting to promote social support, but their results must be regarded as preliminary.

The present study investigated the influence of social support on treatment compliance among hypertensive subjects in a poor urban community in south-west Nigeria. A previous study^13^ of the factors associated with hypertension treatment compliance in this community noted that having social support was associated with treatment compliance. In the present study, we proceeded to explore the issue of social support further by (1) exploring the relationship between social support and good compliance with treatment for hypertension, and (2) identifying factors associated with receiving social support from family and/or friends.

## Methods

This descriptive study was conducted in Idikan community, Ibadan, a city in the south-western part of Nigeria, as part of a larger community-based study of the sociological aspects of hypertension. Ethical approval for the study was obtained from the joint University of Ibadan–University College Hospital ethical committee.

Idikan is located in the indigenous part of the city of Ibadan and has a population of 15 042.^14^ The health facilities in the community include an outreach clinic run by the Department of Preventive Medicine and Primary Care of the University of Ibadan, four private clinics and a small dental clinic run by the Dental Centre of University College Hospital (UCH). There are over 150 registered patent medicine stores in the area. There are three traditional healing homes, and they are all accessible to members of the community.

The study was a descriptive, community-based, quantitative study of hypertensive subjects aged 25 years and above who were residents of Idikan community. Previous studies in the community^15^,^16^ had conducted household screening for hypertension, which facilitated the identification of hypertensive subjects in the community. The subjects for this study were selected from a list of known hypertensive subjects residing in the community that was developed from one such previous hypertension study and updated for the present study during home visits.

Four hundred and forty hypertensive subjects were enrolled using a consecutive sampling method. After obtaining informed consent, subjects were administered a semi-structured questionnaire that had items on several issues, including healthcare seeking for hypertension, their beliefs about hypertension, compliance with treatment, and availability of social support (from family and friends).

Social support for compliance was assessed in the structured questionnaire using the questions: (1) Do you normally seek financial support from family members for your hypertension? (2) How concerned are your family members about your hypertension? (3) How interested are your friends in talking with you about your hypertension? (4) How helpful are your family in reminding you to take your hypertension medication? (5) How helpful are your friends in reminding you to take your hypertension medication? Social support from family members or friends was defined as reporting support from family as ‘helpful’ or ‘very helpful’ in being concerned about respondents’ hypertension (non-directive support), and reminding of medication (directive support).

As previously described^13^ compliance was defined using the question on how frequently people missed taking their medication. The use of compliance as a variable was defined by first scaling compliance as ‘good’ or ‘high compliance’ (where the respondent ‘never misses’ or ‘rarely misses’ taking his/her medication doses), ‘medium compliance’ (where the respondent ‘sometimes misses’ taking medication) and ‘poor’ or ‘low compliance’ (where the respondent ‘regularly misses’ or ‘fairly regularly misses’ taking the medication. Since the desired goal of treatment for hypertension is that the patient complies with taking medication in order to control the high blood pressure, we focused on ‘good compliance’ (where the respondent ‘never misses’ or ‘rarely misses’ taking his/her medication doses) as the main outcome variable to evaluate compliance in this study.

## Statistical analysis

Management and analysis of the survey questionnaire data was done using SPSS version 11 (SPSS Inc, Chicago, USA). Frequencies of the responses to the questions were computed and presented as percentages. Association between categorical variables was tested using the Chi-square test.

## Results

The 440 respondents comprised 65.2% women and 34.8% men. About half (51.1%) of the respondents had no formal education and half were traders. The ages of respondents ranged from 25 to 90 years, with a mean age of 60 (SD 12) years. Most (71%) of the respondents were married (Table 1). Most (70.0%) of the respondents knew about their hypertensive condition only when they were invited to participate in a research study, during which their blood pressure was measured, while 23.0% of the respondents found out that they were hypertensive when they were ill with some other ailment and went to hospital for treatment. The most common perceived causes of hypertension were anxiety (35.7%) and stress (25.2%), followed by mental illness (7.5%) and ‘unhappiness’ (5.5%).

**Table 1:** Demographic characteristics of respondents

*Characteristic*	*Number*	*Percentage*
Smoking	15	3.4
Alcohol use		
Beer	10	2.3
Wine	13	3.0
Whisky	10	2.3
Other liquor	8	1.8
Religion		
Islam	270	61.4
Christianity	169	38.4
Traditional	1	0.2
Ethnic group		
Yoruba	434	98.6
Ibo	5	1.2
Isoko	1	0.2
Educational level		
No formal education	225	51.1
Primary education	86	19.5
Secondary education	49	11.1
Post-secondary education	77	17.5
Other (Arabic school)	3	0.7
Occupation		
Trading	220	50.0
Artisan	49	11.1
Teaching/civil servant	43	9.8
Retired/not working	113	25.7
Religious teachers	15	3.4
Taking antihypertensive medication	257	58.5

The majority (77.5%) of the respondents claimed they complied with keeping their follow-up clinic appointments every time, and 46% said they were on medication at the time of the study. Roughly one-half (50.7%) of respondents had good compliance with treatment as they claimed to be taking their medication regularly, whereas 41.5% had poor compliance at different levels, ranging from regularly missing taking their medication to rarely taking their medication.

## Social support and treatment compliance

Having a family member with hypertension was significantly associated (*p* = 0.038) with compliance in general with 49.3% of those who said ‘yes’ versus 61.7% of those who answered ‘no’. Overall, 85 (19.3%) of the respondents reported that family members were very concerned about their hypertension while 329 (74.8%) said family members were extremely concerned about their hypertension. Also, 89 (20.2%) and 322 (73.2%), respectively, reported that family members were very helpful or extremely helpful in reminding them about taking their medication.

Regarding support from friends, 116 (26.4%) of respondents reported that friends were very concerned about their hypertension while 127 (28.9%) said family members were extremely concerned about their hypertension. Ninety-one (20.7%) and 150 (34.1%) respectively reported that family members were very helpful or extremely helpful in reminding them about taking their medication.

Both having a family member with hypertension and having a family member who had suffered complications were not associated with good compliance. On the other hand, having friends who were concerned about the respondent’s hypertension or who were helpful in reminding the respondent about taking medication were associated with good compliance. A higher proportion of those whose friends were very concerned about their hypertension reported good compliance than those who did not get such support from their friends (*p* ≤ 0.0001). Similarly, a higher proportion of respondents whose friends were very helpful in reminding them about their hypertension medication reported good compliance than those who did not get such support from their friends (*p* ≤ 0.0001) (Table 2).

**Table 2:** The association between social support and good treatment compliance in hypertension

		*Good compliance*		
*Variable*	*Response*	*n (%)*	*χ2*	*p*
Has a family member with hypertension	Yes	36 (49.3)	6.233	0.044*
	No	206 (61.7)		
	Don’t know	15 (45.5)		
Has a family member who has serious health problems from hypertension	Yes	12 (36.4)	9.064	0.011*
	No	230 (61.2)		
	Don’t know	15 (48.4)		
Family members concerned about respondents hypertension	Not very	10 (47.6)	4.128	0.248
	Don’t know	43 (50.6)		
	Very	201 (61.1)		
	Extremely	3 (60.0)		
Family members helpful in reminding about medication	Not very	12 (54.6)	5.132	0.162
	Don’t know	43 (48.3)		
	Very	198 (61.5)		
	Extremely	4 (57.1)		
Friends concerned about respondent’s hypertension	Not very	95 (49.0)	35.700	< 0.0001*
	Don’t know	59 (50.9)		
	Very	102 (80.3)		
	Extremely	1 (33.3)		
Friends helpful in reminding about medication	Not very	96 (49.2)	41.738	< 0.0001*
	Don’t know	40 (44.0)		
	Very	119 (79.3)		
	Extremely	2 (50.0)		

*p < 0.05.

## Factors associated with receiving social support

Having found a significant association between some aspects of social support (from friends) and good treatment compliance, we investigated socio-demographic factors influencing receiving social support. As shown in Table 3, a higher proportion of older respondents (> 55 years) rather than younger respondents reported receiving social support from family (*p* < 0.0001). Gender was not significantly associated with respondents getting social support. On the other hand, there was a significant association between those who were currently married at the time of the study and support from family (*p* = 0.0006) and support from friends (*p* = 0.009).

**Table 3:** Social demographic characteristics and receiving social support among Nigerians with hypertension

*Characteristic*	*n*	*Support^+^ from family n (%)*	*χ2 (*p*-value)*	*Support^+^ from friends n (%)*	*χ2 (*p*-value)*
Age group					
25–55 years	171	101 (59.1)	37.28	58 (33.9)	0.11
> 55 years	223	223 (85.1)	(*p* ≤ 0.001)*	93 (35.5)	(*p* = 0.736)
Gender					
Male	137	99 (72.3)	1.34	56 (40.9)	3.17
Female	275	213 (77.5)	(*p* = 0.247)	88 (32.0)	(*p* = 0.075)
Current marital status					
Unmarried	128	219 (70.2)	11.93	121 (38.8)	6.74
Married	312	110 (85.9)	(*p* = 0.0006)*	33 (25.8)	(*p* = 0.009)*
Religion					
Islam	270	217 (80.1)	10.51	108 (39.9)	7.30
Christianity	169	112 (66.3)	(*p* = 0.001)*	46 (27.2)	(*p* = 0.007)*
Educational level					
No formal education	225	190 (84.4)	22.83	77 (34.2)	0.12
Some education	215	139 (64.7)	(*p* ≤ 0.001)*	77 (35.8)	(*p* = 0.726)

^+^Support defined as being ‘helpful’ or ‘very helpful’ in reminding of medication. *p < 0.05.

It is of interest also to note that both religion and educational level of respondents were significantly associated with getting social support from both family and friends. A higher proportion of respondents of the Islamic faith (in contrast to Christians) received social support from family and friends, respectively, while respondents with no formal education (in contrast to those with some education) received social support from members of their families.

## Discussion

Social support is a construct that describes the structure of a person’s social environment and the tangible, instrumental and emotional resources the social environment provides. A wealth of data, particularly from large, long-term, observational studies, has shown that higher levels of social support, whether measured by instrumental, tangible or emotional indices, are associated with reduced cardiovascular morbidity and mortality.^9^,^17^-^19^ The disease-related protective effects of social support were first described in the 1970s.^20^ From that time, there has been great interest in the relationship of social support to health, and in particular to cardiovascular disease.^21^ However, there is scarcity of such studies from Africa.

The issue of social support for health issues in African societies warrants close study, given some of the characteristics of these societies. For example, there is an emphasis on the family and community rather than the individual, and many individuals live in extended (rather than nuclear) family set-ups. This often means that an individual’s problems (including health issues) are not his/hers alone but that of the family. On the other hand, individuals may conceal medical diagnoses for various reasons (e.g. stigma, fear of being considered ‘different’ or of the family being perceived as ‘tainted’ or cursed), which means family members and friends may be unaware and cannot provide support.

In Ushie and Jegede’s study^22^ on the paradox of family support, concerns of tuberculosis-infected HIV patients about involving family and friends in their treatment reported that family support was *expressly* seen by participants as central to medication adherence but one of the main drawbacks to its maximal utilisation was fear of condemnation and stigma from family members and friends, and from the family as a whole, which makes people with HIV and/or TB hide their status.

Chronic illnesses that require life-long treatment (such as hypertension and diabetes) pose unique challenges in such a context, not least of which is the need to maintain the motivation to adhere to treatment for many years. The need to understand social support in such a context was the primary motivation of this study.

In the present study, those who had support from friends or family members (concerned about their illness, giving reminders about medication) showed better treatment compliance than those who did not, although this difference was greatest for those who had the support of friends. This is an important finding and is consistent with what has been reported for multiple chronic diseases in several parts of the world.^23^,^24^ Interestingly, the evidence from this study shows that support from friends is a stronger factor influencing good compliance than support from family members.

By contrast, Marin-Reyes and Rodriguez-Moran^11^ found that compliance with hypertensive treatment was directly linked to the support of family members. The findings of the present study may be a reflection of the fact that most people in this urban community (and in cities in general) talk and interact more with their friends than with their family members who do not live nearby. In this regard, it would be important to study people who live in rural areas where living in extended-family and multi-generational households is more common. Another explanation may be that those with hypertension are more likely to discuss their health problems with their friends than with family members, thereby inadvertently limiting the support they could receive from the latter.

Given the role played by social support in compliance with hypertension treatment in this community, it was instructive to attempt to identify the factors associated with receiving such support. While a specific subset of factors (demographic factors) was explored in this study, age, marital status, religion and educational level were each associated with receiving social support. Each of these factors is noteworthy. However, it is difficult to evaluate how demographic factors interact with the larger set of factors known to be associated with social support. For example, it is known that marked cultural differences exist in the types and effectiveness of social support, as well as in how people use their support networks.^23^ These cultural differences may underlie some or most of the apparent relationships with demographic factors observed in this study.

The findings of this study suggest ways in which social support could be used in the treatment of hypertension in this community. First, it would seem that adding social support to treatment guidelines could improve awareness by healthcare providers of this important component of treatment compliance. Second, teaching health providers how to explore and utilise their patients’ social support networks may help to improve treatment compliance. Third, exploring the use of existing social networks (e.g. peer groups, cultural groups, religious groups) in this and similar communities may impact on how social support can be leveraged to improve health behaviours.

To our knowledge, this is the first study focused on social support with regard to treatment compliance in hypertension or cardiovascular disease in Nigeria. The strengths of the study include a large sample size, focus on a single non-communicable condition, which limits heterogeneity from differing diseases and their treatments, and a community-based design, which better permits generalisation than a hospital-based design. Limitations include a cross-sectional design, which does not permit identification of cause and effect, and the use of selfreport measures.

However, this was an exploratory study and more studies are needed to confirm and extend the findings. Such studies should be designed to ameliorate or overcome the limitations of the present study, including the use of more comprehensive and validated social support assessment tools, collecting more variables on each subject, inclusion of qualitative methods, and the development of mechanisms/models of social support and their role in health behaviours.

## Conclusion

We concluded that social support is strongly associated with hypertension treatment compliance in this community in southwest Nigeria. These findings suggest a need for exploring the promotion of social support as a useful tool in chronic disease treatment programmes.

## References

[1] House JS, Landis KR, Umberson D (1988). Social relationships and health science.. Science.

[2] Olsen O (1993). Impact of social network on cardiovascular mortality in middle aged Danish men.. J Epidemiol Commun Health.

[3] Seeman TE, Berkman LF, Kohout F, Lacroix A, Glynn R, Blazer D (1993). Intercommunity variation in the association between social ties and mortality in the elderly: a comparative analysis of three communities.. Ann Epidemiol.

[4] Vogt TM, Mullooly JP, Ernst D, Pope CR, Hollis JF (1992). Social networks as predictors of ischemic heart disease, cancer, stroke and hypertension: incidence, survival and mortality.. J Clin Epidemiol.

[5] Welin L, Larsson B, Svardsudd K (1992). Social network and activities in relation to mortality from cardiovascular diseases, cancer and other causes: a 12 year follow up of the study of men born in 1913 and 1923.. J Epidemiol Commun Health.

[6] Berkman LF, Glass T, Berkman LF, Kawachi I (2000). Social integration, social networks, and health.. Social Epidemiology..

[7] Kaplan RM, Toshima MT, Sarason BR, Sarason IG, Pierce GR (1990). The functional effects of social relationships on chronic illnesses and disability.. Social Support: An Interactional View..

[8] Aalto AM, Uutela A, Aro AR (1997). Health related quality of life among insulin-dependent diabetics: Disease-related and psychosocial correlates.. Patient Education Counsel.

[9] Rozanski A, Blumenthal JA, Kaplan J (1999). Impact of psychological factors on the pathogenesis of cardiovascular disease and implications for therapy.. Circulation.

[10] Blazer DG (1982). Social Support and mortality in an elderly community population.. Am J Epidemiol.

[11] Marin-Reyes F, Rodriguez-Moran M (2001). Family support of treatment compliance in essential arterial hypertension.. Salud Publica Mex.

[12] Bharati V, Mittal MD, Ajay K, Singh (2010). Hypertension in the developing world: challenges and opportunities.. Am J Kidney Dis.

[13] Osamor PE, Owumi BE (2011). Factors associated with treatment compliance in hypertension in southwest Nigeria.. J Health Popul Nutr.

[14] (2009). Nigeria Demographic and Health Survey.

[15] Lawoyin TO, Asuzu MC, Kaufman J, Rotimi C, Owoaje E, Johnson L, Cooper R (2002). Prevalence of cardiovascular risk factors in an African, urban inner city community.. West Afr J Med.

[16] Adeyemo AA, Omotade OO, Rotimi CN, Luke AH, Tayo BO, Cooper RS (2002). Heritability of blood pressure in Nigerian families.. J Hypertens.

[17] Burg MM, Barefoot J, Berkman L, Catellier D, Czajkowski S, Saab P (2005). Low perceived social support and post-myocardial infarction prognosis in the enhancing recovery in coronary heart disease clinical trial: the effects of treatment.. Psychosomat Med.

[18] Cohen S (1988). Psychosocial models of the role of social support in the etiology of physical disease.. Health Psychol.

[19] Rozanski A, Blumenthal JA, Davidson KW, Saab PG, Kubzansky L (2005). The epidemiology, pathophysiology, and management of psychosocial risk factors in cardiac practice: the emerging field of behavioral cardiology.. J Am Coll Cardiol.

[20] Cobb S (1976). Presidential address – 1976. Social support as a moderator of life stress.. Psychosomat Med.

[21] Lett HS, Blumenthal JA, Babyak MA, Strauman TJ, Robins C, Sherwood A (2005). Social support and coronary heart disease: epidemiologic evidence and implications for treatment.. Psychosomat Med.

[22] Ushie BA, Jegede SA (2012). The paradox of family support: concerns of tuberculosis-infected HIV patients about involving family and friends in their treatment.. Aids Patient Care STDs.

[23] Oxman TE, Hull JG (2001). Social support and treatment response in older depressed primary care patients.. J Gerontol Psycholog Sci.

[24] Strogatz DS, James SA (1986). Social support and hypertension among blacks and whites in a rural, southern community.. Am J Epidemiol.

